# Silent Intrusion: Left Atrial Myxoma in an Afro-Caribbean Woman

**DOI:** 10.7759/cureus.104184

**Published:** 2026-02-24

**Authors:** Roslyn Alfred-Demas, Jana-Abbi Tanner, Jannike Frank-Edobor, Je Vaughn Clarke, Jodi Benjamin

**Affiliations:** 1 Internal Medicine, Tobago Regional Health Authority, Scarborough, TTO; 2 Internal Medicine, Scarborough General Hospital, Scarborough, TTO

**Keywords:** atrial mass, cardiac tumor, left atrial myxoma, myxoma, myxomatous tissue

## Abstract

Although cardiac myxomas are benign tumors, they can be associated with significant symptoms and serious embolic complications. Timely investigations and diagnosis can prevent a myriad of complications. Definitive treatment, in the form of surgical excision, can prevent life-threatening embolization. They are the most common benign cardiac tumors, but they remain relatively rare and can mimic inflammatory disorders. Therefore, a high index of suspicion is warranted when evaluating certain symptoms. This is the case report of a middle-aged, Afro-Caribbean woman who presented with mild dyspnea. Further investigations led to the finding of a silent left atrial myxoma. Caribbean data on incidence and prevalence are sparse. Education regarding presentation and indications for anticoagulation is useful and relevant, as treatment is evolving. A multidisciplinary approach to managing these patients is essential.

## Introduction

Myxomas are the most common benign primary cardiac tumor [1]. Like Grebenc et al., Keeling et al. also reported that they are relatively rare, with a prevalence of 0.03% in the general population and an incidence of 0.5-1 case per million individuals [2,3]. Data from the Caribbean are sparse, but a study conducted in Puerto Rico by Bou et al. reported 55 cases over a 14-year period in a population of three million [4]. In that study, the median age was 52 years, and cases were predominantly women. Most cardiac myxomas occur sporadically, but a minority may be part of the familial, inherited Carney complex. This autosomal dominant condition and its subsets (Lentigines, Atrial Myxomas, and Blue nevi, and Nevi, Atrial myxoma, Myxoid neurofibromas, and Ephelides syndromes) include cardiac and skin myxomas, skin hyperpigmentation, and endocrinopathies, as described by Sandrini and Stratakis [5]. These types present at a younger age and are part of the multiple endocrine neoplasia syndrome [1]. Clinical presentation ranges from asymptomatic to symptomatic and may mimic autoimmune conditions; therefore, evidence-based data emphasize the importance of physician education to recognize clinical cues that warrant cardiac imaging [6]. Recent studies conclude that although surgical removal is the definitive treatment, a multidisciplinary approach is required for best medical practice [5,6].

## Case presentation

We describe the case of a 54-year-old female patient who was referred after COVID-19 because of an irregular pulse. She had no significant past medical history or comorbidities. She was a nonsmoker and had a relatively sedentary lifestyle. Family history was unremarkable for cardiac pathology or sudden cardiac death.

She was previously treated at the COVID-19 isolation facility three months prior to her presentation to the medical outpatient clinic. She was treated for moderate COVID-19 pneumonia and a urinary tract infection. She complained of mild shortness of breath. She was referred for evaluation by an internist and for a transthoracic echocardiogram. The patient had no other symptoms. She reported a gynecological history of uterine fibroids and an endometrial polyp, with a past surgical history of polypectomy. Of note, she was on no chronic medications.

**Figure 1:**
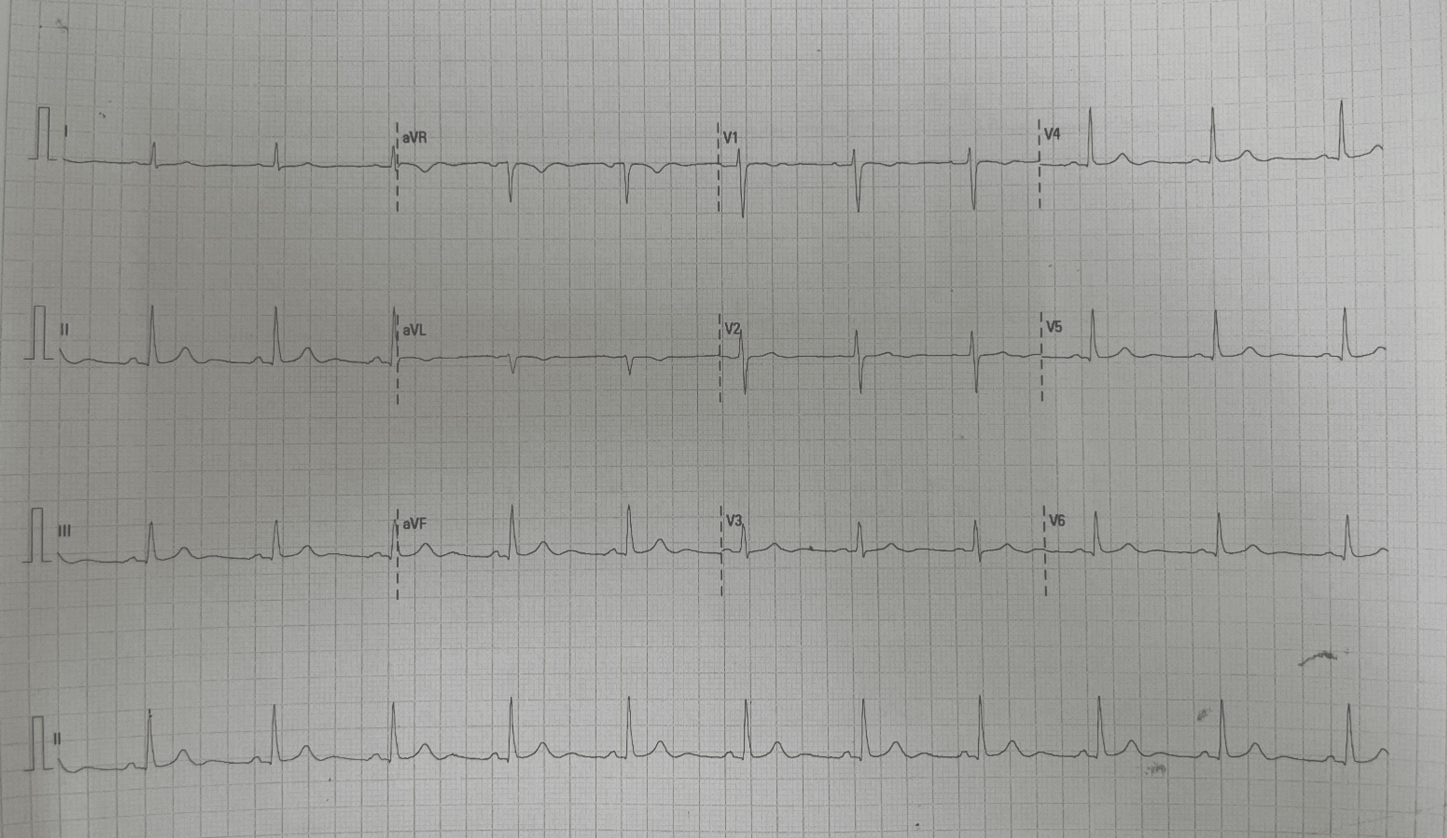
Electrocardiogram of the patient on the first presentation showing sinus rhythm aVR: augmented vector right; aVL: augmented vector left; aVF: augmented vector foot

On examination, the patient was mildly obese and normotensive, and her pulse was mildly irregular intermittently with no murmurs on cardiac auscultation. There was no organomegaly and no neurological deficits. Her electrocardiogram showed sinus rhythm (Figure 1). The hematological, renal, and thyroid function blood investigations were normal. Transthoracic echocardiogram showed a mobile mass attached to the interatrial septum with a wide base, extending to the mitral annulus and mildly protruding into the left ventricle during systole. It measured 29 mm wide by 37 mm high. Her ejection fraction was normal (68%). The left atrial size was 34 mm. The mitral valve was of normal structure and function, with mildly turbulent transmittal flow (but no significant gradient). Contrast-enhanced computed tomography (CECT) of her chest suggested that this was indeed an atrial tumor and not a thrombus. The images of the CECT and echocardiogram are not available. No medication was initiated.

**Figure 2:**
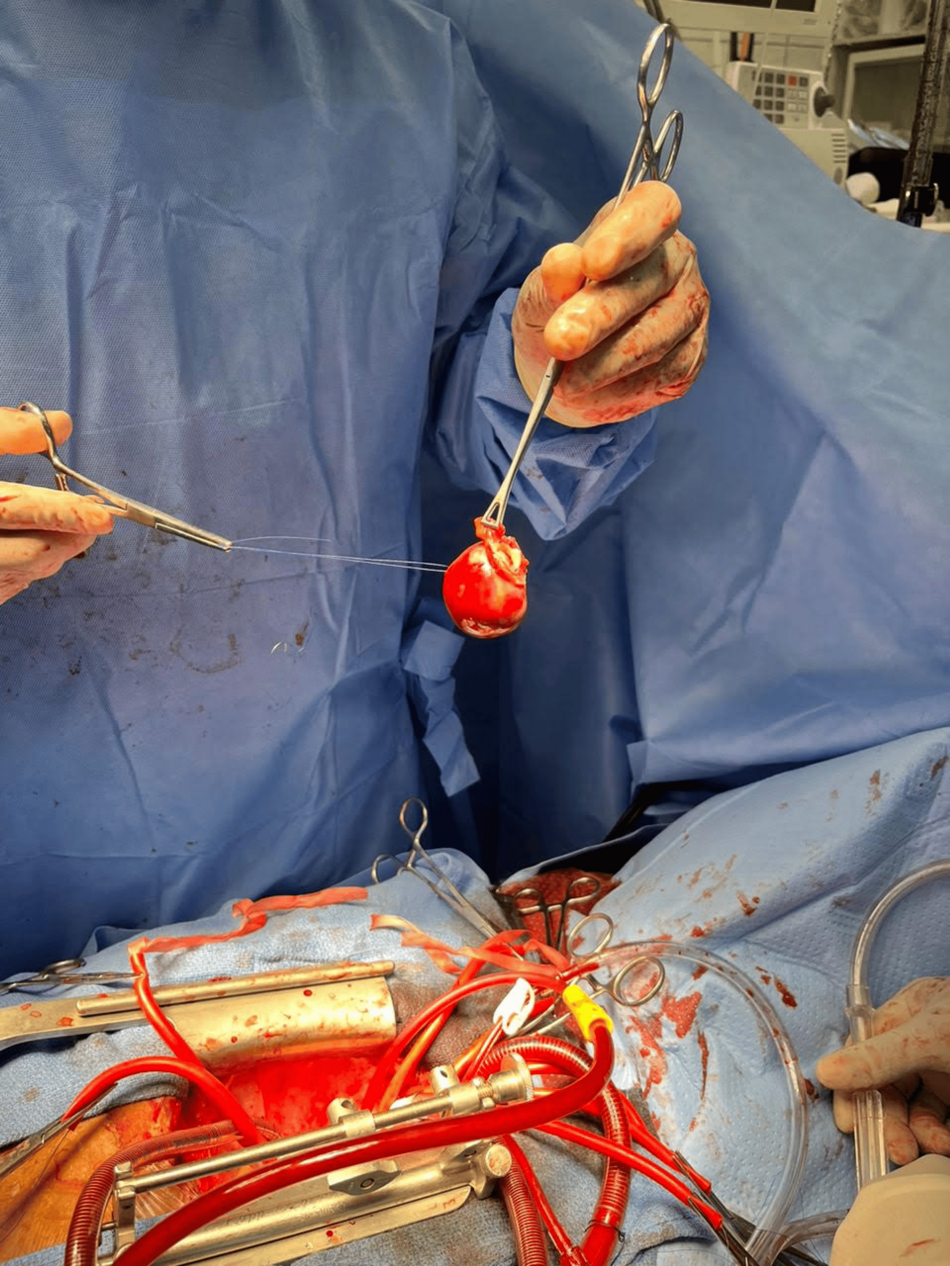
Left atrial myxoma (isolated and removed intraoperatively)

She was referred to the cardiothoracic surgical clinic, and she was scheduled for surgical removal, which was done within three months. The patient underwent open cardiothoracic surgery with complete surgical excision of the atrial myxoma (Figure 2). The histology report revealed a polypoid myxoid matrix of polygonal cells with inconspicuous eosinophilic cytoplasm and a few interspersed macrophages. No malignant features were noted, and the mass was confirmed as an atrial myxoma. Pathology images are not available for review. A repeat transthoracic echocardiogram a year later showed no recurrence. The patient has been doing well clinically. No medical therapy was initiated.

## Discussion

Most cardiac myxomas occur in the cardiac atria, particularly on the left side (75%), and their presentation can range from asymptomatic to a wide range of symptoms, according to Velez Torres et al. [6]. They are described as “functionally malignant” because they are at high risk of embolization and fatal complications, such as sudden cardiac death [7]. A clinical triad of intracardiac obstruction, systemic or pulmonary embolization, and constitutional symptoms is characteristic of cardiac myxomas. A low-pitched diastolic “tumor plop” sound may be heard after S2 when the pedunculated left atrial tumor bounces off the mitral valve or ventricular wall during rapid diastolic filling. The tumor's motion halts abruptly upon impact with the ventricular wall or taut mitral valve leaflets. This is distinct from the high-pitched sound of rheumatic mitral valve disease. Left-sided tumors may also produce a murmur of functional mitral stenosis. Atrial fibrillation may also be a presenting feature [4,7]. Right-sided tumors may produce a murmur similar to that of tricuspid stenosis, which is louder on inspiration [7].

Echocardiography is the diagnostic modality of choice, whether transesophageal or transthoracic [8,9]. Although surgery is curative, it should be performed promptly to prevent complications such as congestive heart failure, thromboembolic events, arrhythmias, valvular defects, and infections [10]. Surgical excision is the definitive treatment for atrial myxomas and can be performed via traditional open-heart surgery or a mini thoracotomy [8]. Recently, robotic surgery, tumor embolization, and radiofrequency ablation have become minimally invasive options [8]. The choice between open and minimally invasive approaches may depend on tumor size and complexity, patient comorbidities, and surgical expertise. In the study by Ashinze et al., patients for open surgery had a longer hospital stay and a higher incidence of postoperative infections, such as mediastinitis [8]. Minimally invasive may not be the choice for larger and more complex atrial myxomas, but the shorter hospital stays and lower risk of postoperative infections will offer advantages.

Anticoagulation preoperatively is controversial, as stated in some reports [11]. The controversy results from the decision to anticoagulate patients with proven systemic emboli on transesophageal echocardiographic imaging and who have had an ischemic stroke related directly to atrial myxomas, while trying to minimize the waiting time for surgical excision [11]. Benefits must outweigh risks when such clinical decisions are required. Cases of severe neurological deficits requiring preoperative anticoagulation before excision in patients at low risk of intracerebral bleed have been reported [11].

Independent prognostic factors after resection include late-onset atrial fibrillation, age, increased left atrial diameter, and the need for mitral valve surgery [12]. The frequency of echocardiogram surveillance is not well documented in many studies [13]. Risk for malignancy increases in those with the familial Carney complex, and this can also impact surveillance. Postoperative surveillance is essential [13]. There is no specific protocol or recommendation regarding the frequency of echocardiographic surveillance. The importance of multidisciplinary surveillance is evident as well.

## Conclusions

Atrial myxomas are the most frequent benign cardiac tumors and could present with a wide range of clinical manifestations. Our patient presented with dyspnea, a quite common symptom of intracardiac obstruction. A prompt multidisciplinary approach is essential to ensure timely and curative surgical excision. The case discussed was seen by the general internist and cardiologist as well as the cardiothoracic surgeon. As physicians, due diligence is required and should be based on clinical history and examination findings. Follow-up surveillance and the frequency of postoperative monitoring for atrial myxomas may be decided on a case-by-case basis, taking into account the risk of recurrence. Our patient did have an echocardiogram a year after excision and remains asymptomatic. There is no specific postoperative surveillance protocol, but one should be cautious of symptoms of the clinical triad of intracardiac obstruction, systemic or pulmonary embolization, and constitutional symptoms is characteristic of cardiac myxomas.
